# Supplementary material for: Ultrasound evaluation of vagus nerve cross-sectional area in a community-dwelling elderly Japanese cohort
Source: PLoS One. 2023 May 18;18(5):e0280661. doi: 10.1371/journal.pone.0280661 (PMC10194966; doi:10.1371/journal.pone.0280661)
Supplement: S1 Checklist — (DOCX) [file pone.0280661.s001.docx]

STROBE Statement—checklist of items that should be included in reports of observational studies

|  | Item No. | Recommendation | Page  No. | Relevant text from manuscript |
| --- | --- | --- | --- | --- |
| **Title and abstract** | 1 | (*a*) Indicate the study’s design with a commonly used term in the title or the abstract | 1 | Ultrasound evaluation of vagus nerve cross-sectional area in a community-dwelling elderly Japanese cohort |
|  |  | (*b*) Provide in the abstract an informative and balanced summary of what was done and what was found | 3,4 | We have reported reference VN CSA values for community-dwelling elderly Japanese individuals. We showed that the CSA of the VN on the left side was positively associated with a history of convulsive seizure and BMI and inversely associated with a history of head injury. |
| Introduction | | | |  |
| Background/rationale | 2 | Explain the scientific background and rationale for the investigation being reported | 5,6 | As ultrasound of the peripheral nerves is being increasingly used to conduct structural assessments in various diseases, well-defined references values for the CSA of the VN are essential. Data regarding the VN in older East Asian and specifically Japanese populations are lacking. |
| Objectives | 3 | State specific objectives, including any prespecified hypotheses | 6 | This study aimed to determine reference values for the CSA of the VN in community-dwelling elderly Japanese individuals and to identify any medical history and/or lifestyle factors associated with these values. |
| Methods | | | |  |
| Study design | 4 | Present key elements of study design early in the paper | 6,7 |  |
| Setting | 5 | Describe the setting, locations, and relevant dates, including periods of recruitment, exposure, follow-up, and data collection | 7,8 | This cross-sectional study of the VN was conducted from October 2021 to February 2022. |
| Participants | 6 | (*a*) *Cohort study*—Give the eligibility criteria, and the sources and methods of selection of participants. Describe methods of follow-up  *Case-control study*—Give the eligibility criteria, and the sources and methods of case ascertainment and control selection. Give the rationale for the choice of cases and controls  *Cross-sectional study*—Give the eligibility criteria, and the sources and methods of selection of participants | 6,7 | The Japan Prospective Studies Collaboration for Aging and Dementia (JPSC-AD) is a multicenter prospective cohort study of dementia that surveyed more than 10,000 community-dwelling elderly persons aged 65 years or older at eight study sites in Japan. Among the participants in the JPSC-AD, only those who enrolled in the Yahaba Active Aging and Healthy Brain (YAHABA) study for the residents of Yahaba Town, Japan were included in the present study. The YAHABA study is a community-based prospective cohort study that was established in 2016 to clarify the risk factors and etiology of dementia, cerebrovascular diseases, and movement disorders in older adults. |
|  |  | (*b*) *Cohort study*—For matched studies, give matching criteria and number of exposed and unexposed  *Case-control study*—For matched studies, give matching criteria and the number of controls per case | N/A |  |
| Variables | 7 | Clearly define all outcomes, exposures, predictors, potential confounders, and effect modifiers. Give diagnostic criteria, if applicable | 9,10 | Factors that were significant in the simple linear regression analysis were selected for evaluation as independent parameters by multivariate analysis. For the multivariable linear regression analysis, the World Health Organization BMI classification was used to categorize the participants as underweight (BMI < 18.5 kg/m^2^), normal (BMI 18.5–24.9 kg/m^2^), overweight (BMI 25.0–29.9 kg/m^2^), or obese (BMI ≥ 30 kg/m^2^). |
| Data sources/ measurement | 8* | For each variable of interest, give sources of data and details of methods of assessment (measurement). Describe comparability of assessment methods if there is more than one group | 7,8,9 | All information on medical history was based on the self-reports of the study participants. Height, weight, body mass index (BMI), and blood pressure were measured. Blood pressure was measured three times using an automated sphygmomanometer with the study participants in a seated position after at least 5 minutes of rest; the average of the three measurements was calculated. Height and weight were measured while the participants were wearing light clothing without shoes, and BMI was calculated. Casual blood samples were drawn from the antecubital vein of all participants to test the fasting blood glucose and hemoglobin A1c concentrations. Participants with a fasting blood glucose concentration ≥ 126 mg/dL (7.0 mmol/L) or hemoglobin A1c concentration ≥ 6.5% or who reported a history of DM were considered to have DM. |
| Bias | 9 | Describe any efforts to address potential sources of bias | N/A |  |
| Study size | 10 | Explain how the study size was arrived at | N/A |  |

Continued on next page

| Quantitative variables | 11 | Explain how quantitative variables were handled in the analyses. If applicable, describe which groupings were chosen and why | 9 | Simple linear regression analysis was used to evaluate the associations between the CSA of the VN and the individual background characteristics of the participants. |
| --- | --- | --- | --- | --- |
| Statistical methods | 12 | (*a*) Describe all statistical methods, including those used to control for confounding | 9 | Simple linear regression analysis was used to evaluate the associations between the CSA of the VN and the individual background characteristics of the participants. Factors that were significant in the simple linear regression analysis were selected for evaluation as independent parameters by multivariate analysis. |
|  |  | (*b*) Describe any methods used to examine subgroups and interactions | N/A |  |
|  |  | (*c*) Explain how missing data were addressed | N/A |  |
|  |  | (*d*) *Cohort study*—If applicable, explain how loss to follow-up was addressed  *Case-control study*—If applicable, explain how matching of cases and controls was addressed  *Cross-sectional study*—If applicable, describe analytical methods taking account of sampling strategy | N/A |  |
|  |  | (*e*) Describe any sensitivity analyses | N/A |  |
| Results | | | | |
| Participants | 13* | (a) Report numbers of individuals at each stage of study—eg numbers potentially eligible, examined for eligibility, confirmed eligible, included in the study, completing follow-up, and analysed | 10 | After excluding 10 subjects who were incapable of providing informed consent, a total of 336 participants were included in this study. |
|  |  | (b) Give reasons for non-participation at each stage | 10 | After excluding 10 subjects who were incapable of providing informed consent, a total of 336 participants were included in this study. |
|  |  | (c) Consider use of a flow diagram | N/A |  |
| Descriptive data | 14* | (a) Give characteristics of study participants (eg demographic, clinical, social) and information on exposures and potential confounders | 10,11 | The characteristics of the participants are shown in Table 1. |
|  |  | (b) Indicate number of participants with missing data for each variable of interest | N/A |  |
|  |  | (c) *Cohort study*—Summarise follow-up time (eg, average and total amount) | N/A |  |
| Outcome data | 15* | *Cohort study*—Report numbers of outcome events or summary measures over time | N/A |  |
|  |  | *Case-control study—*Report numbers in each exposure category, or summary measures of exposure | N/A |  |
|  |  | *Cross-sectional study—*Report numbers of outcome events or summary measures | N/A |  |
| Main results | 16 | (*a*) Give unadjusted estimates and, if applicable, confounder-adjusted estimates and their precision (eg, 95% confidence interval). Make clear which confounders were adjusted for and why they were included | 13,14,15,16 | We conducted simple linear regression analysis to determine the relationship between the background of the participants and the CSA of the VN on the left and right sides (Table 2). Multivariable linear regression analysis with adjustments for age, sex, BMI, AF, DM, head injury, convulsion, hypertension, and smoking habit showed that history of head injury (β = -0.15, p < .01), history of convulsion (β = .19, p < .01), and BMI (β = .30, p < .01) were independently associated with the CSA of the VN on the left side, while none of the variables were significantly associated with the CSA of the VN on the right side (Table 3). |
|  |  | (*b*) Report category boundaries when continuous variables were categorized | N/A |  |
|  |  | (*c*) If relevant, consider translating estimates of relative risk into absolute risk for a meaningful time period | N/A |  |

Continued on next page

| Other analyses | 17 | Report other analyses done—eg analyses of subgroups and interactions, and sensitivity analyses | N/A |  |
| --- | --- | --- | --- | --- |
| Discussion | | | | |
| Key results | 18 | Summarise key results with reference to study objectives | 16 | The present study provides reference values for the CSA of the VN in Japanese community-dwelling individuals aged 65 years and older. Moreover, multivariable linear regression analysis revealed that the CSA of the VN on the left side was positively associated with a history of convulsion and higher BMI and inversely associated with a history of head injury. In contrast, there were no independent associations between any of the assessed variables and the CSA of the VN on the right side. |
| Limitations | 19 | Discuss limitations of the study, taking into account sources of potential bias or imprecision. Discuss both direction and magnitude of any potential bias | 22 | The present study had some limitations. First, the accuracy of the CSA measurements was not examined. Five medical laboratory technicians measured the CSA of the VN in our study cohort, but intra- and inter-observer reliabilities could not be determined as each CSA was measured only once by a single technician. Second, as this was a cross-sectional study, we could not address causal relationships between the variables and the CSA of the VN. Third, the resolution of the ultrasound probes was relatively low for the measurement of nerves; future studies should use probes with resolutions of 15 MHz or more. Fourth, as medical history (including previous injuries) was self-reported by the study participants, there was potential for inaccuracies in the information recorded. Finally, in the future, it will be important to evaluate the CSAs of nerves other than the VN in a Japanese cohort, to see if they are also smaller than the values reported for other populations. |
| Interpretation | 20 | Give a cautious overall interpretation of results considering objectives, limitations, multiplicity of analyses, results from similar studies, and other relevant evidence | 22,23 | In conclusion, we determined reference values for the CSA of the VN in a large cohort of community-dwelling elderly Japanese individuals. The CSA of the VN on the left side was positively associated with history of convulsive seizure and BMI and inversely associated with history of head injury. In contrast, there were no independent associations between any of the assessed variables and the CSA on the right side. |
| Generalisability | 21 | Discuss the generalisability (external validity) of the study results | 22,23 | A longitudinal prospective cohort study is warranted to evaluate the causal relationships between the clinical/background factors identified in this study and the CSA of the VN. |
| Other information | |  | | |
| Funding | 22 | Give the source of funding and the role of the funders for the present study and, if applicable, for the original study on which the present article is based | Provided in a separate file. |  |

*Give information separately for cases and controls in case-control studies and, if applicable, for exposed and unexposed groups in cohort and cross-sectional studies.

**Note:** An Explanation and Elaboration article discusses each checklist item and gives methodological background and published examples of transparent reporting. The STROBE checklist is best used in conjunction with this article (freely available on the Web sites of PLoS Medicine at http://www.plosmedicine.org/, Annals of Internal Medicine at http://www.annals.org/, and Epidemiology at http://www.epidem.com/). Information on the STROBE Initiative is available at www.strobe-statement.org.
